# Alveolar echinococcosis in solid organ transplant recipients: a case series from two national cohorts[Fn FN1]

**DOI:** 10.1051/parasite/2023008

**Published:** 2023-03-31

**Authors:** Bastian Marquis, Florent Demonmerot, Carine Richou, Gérard Thiéfin, Laurence Millon, Martine Wallon, Dominique Angèle Vuitton, Anne Grall-Jezequel, Frédéric Grenouillet, Olivier Epaulard, Philippe Gervais, Oriol Manuel, Solange Bresson-Hadni

**Affiliations:** 1 Infectious Diseases Unit, Department of Medicine, University Hospital and University of Lausanne CH-1011 Lausanne Switzerland; 2 Parasitology-Mycology Laboratory, French National Reference Center for Echinococcosis, University Hospital of Besançon and Franche-Comté University F-25030 Besançon France; 3 UMR 6249 CNRS-Franche-Comté University « Chrono-Environnement » F-25030 Besançon France; 4 Liver Diseases Unit, French National Reference Center for Echinococcosis, University Hospital of Besançon and Franche-Comté University F-25030 Besançon France; 5 Hepato-Gastroenterology Department, Reims Champagne Ardennes University and University Hospital of Reims F-5192 Reims France; 6 Parasitology-Mycology Laboratory, University Hospital of Lyon F-69317 Lyon France; 7 French National Reference Center for Echinococcosis and Franche-Comté University F-25030 Besançon France; 8 Nephrology Service, Brest University Hospital Centre F-29609 Brest France; 9 Fungi and Parasite Serology Unit, French National Reference Center for Echinococcosis, University Hospital of Besançon and Franche-Comté University F-25030 Besançon France; 10 Infectious Disease Service, University Hospital of Grenoble F-38700 Grenoble France; 11 Institut Universitaire de Cardiologie et de Pneumologie de Québec, Laval University Quebec City QC G1V 4G5 Canada; 12 Infectious Diseases Unit and Transplantation Center, University Hospital and University of Lausanne CH-1011 Lausanne Switzerland; 13 Visceral Surgery/Liver Transplant Unit, French National Reference Center for Echinococcosis, University Hospital of Besançon and Franche-Comté University F-25030 Besançon France

**Keywords:** Solid-organ transplant, Alveolar echinococcosis, *Echinococcus multilocularis*, Immunosuppression

## Abstract

Alveolar echinococcosis (AE) is a severe parasitic infection caused by the ingestion of *Echinococcus multilocularis* eggs. While higher incidence and faster evolution have been reported in immunosuppressed patients, no studies have been performed specifically on AE in transplant patients. We searched for all *de novo *AE cases diagnosed between January 2008 and August 2018 in solid organ transplant (SOT) recipients included in the Swiss Transplant Cohort Study and the FrancEchino Registry. Eight cases were identified (kidney = 5, lung = 2, heart = 1, liver = 0), half of which were asymptomatic at diagnosis. AE diagnosis was difficult due to the low sensitivity (60%) of the standard screening serology (Em2+) and the frequently atypical radiological presentations. Conversely, *Echinococcus *Western blot retained good diagnostic performances and was positive in all eight cases. Five patients underwent surgery, but complete resection could only be achieved in one case. Moreover, two patients died of peri-operative complications. Albendazole was initiated in seven patients and was well tolerated. Overall, AE regressed in one, stabilized in three, and progressed in one case, and had an overall mortality of 37.5% (3/8 patients). Our data suggest that AE has a higher mortality and a faster clinical course in SOT recipients; they also suggest that the parasitic disease might be due to the reactivation of latent microscopic liver lesions through immune suppression. Western blot serology should be preferred in this population. Finally, surgery should be considered with caution, because of its low success rate and high mortality, and conservative treatment with albendazole is well tolerated.

## Abstract (fr)

L’échinococcose alvéolaire (EA) est une maladie parasitaire grave causée par l’ingestion d’œufs d’*Echinococcus multilocularis*. Bien qu’une plus haute incidence et une évolution plus rapide aient été rapportées chez les patients immunodéprimés, aucune étude n’a été conduite spécifiquement sur cette maladie chez les patients transplantés. Nous avons donc listé tous les cas d’échinococcose alvéolaire apparus *de novo *entre janvier 2008 et août 2018 chez les patients transplantés d’organe solide inclus dans la cohorte Swiss Transplant Cohort Study et le registre FrancEchino. Huit patients ont été identifiés (rein = 5, poumon = 2, cœur = 1, foie = 0), dont la moitié était asymptomatique au moment du diagnostic. Le diagnostic était compliqué par la basse sensibilité (60 %) de la sérologie standard de dépistage (Em2+) et par les présentations radiologiques atypiques des lésions. Les performances diagnostiques du Western Blot n’étaient toutefois pas affectées et ce test était positif chez tous les patients. Sur les cinq patients opérés, une résection complète n’a été possible que dans un cas, tandis que deux patients sont décédés dans les suites de l’opération. L’albendazole a été introduit chez 7 patients et a été bien toléré. Dans l’ensemble, l’EA s’est stabilisée dans 3 cas, a régressé dans un cas et a progressé dans un autre cas, avec une mortalité de 37,5 % (3/8 patients). Nos résultats suggèrent une mortalité plus élevée et une évolution plus rapide de l’EA chez les patients transplantés. Ils suggèrent aussi que la maladie parasitaire pourrait être due à la réactivation de lésions hépatiques microscopiques latentes à la faveur de l’immunosuppression. Le Western Blot devrait être préféré dans cette population. Finalement, la chirurgie devrait être envisagée avec prudence, étant donnés son faible taux de réussite, le nombre élevé de décès peri-opératoires et la bonne tolérance au traitement conservateur par albendazole.

## Introduction

Alveolar echinococcosis (AE) is a parasitic infection caused by the ingestion of eggs of *Echinococcus multilocularis*. The parasite is endemic in several regions of the Northern hemisphere in Europe, Asia, and North America [4, 11, 30]. The annual incidence of AE varies between 0.03 and 1.2 per 100,000 person-year in Europe, and has been increasing in the past few decades [26]. AE evolves similarly to a slow-growing cancer, with a latency period of up to 10 years between the infection and the onset of symptoms [5, 25, 30]. The primary lesion is generally in the liver, but the parasitic lesion often invades surrounding tissues and can metastasize to distant locations, most often the lungs [25]. If AE is left untreated, its mortality can be as high as to 90% at 10 years [30]. Diagnosis relies on radiological imaging and confirmation is obtained by serology. Patients must usually undergo extensive hepatic surgical resection and take an anti-parasitic drug, most often albendazole, for at least 2 years, and for life if non-operated [30].

AE has a higher incidence and a faster course in immunocompromised patients than in the general population [7, 22]. Due to atypical clinical and radiological manifestations and lower sensitivity of the usual serological tests, diagnosis is also more difficult to establish in these patients [7]. AE in solid organ transplant (SOT) recipients has mostly been described in the context of liver transplantation as a last therapeutic option for AE [3, 20]. Such studies showed that recurrence of AE lesions was frequent after transplantation in patients without anti-parasitic treatment; however, albendazole is well tolerated and effective to avoid relapse and/or progression despite the ongoing immunosuppression. Several cases of *de novo* AE in SOT recipients have also been reported and suggest a faster evolution of AE than in other immunocompromised patients [9, 12–14]. No study has, however, been conducted to specifically assess the epidemiology and outcome of AE in this population. We present here a case series including patients with AE from two different nationwide prospective cohorts: the Swiss Transplant Cohort Study (STCS) and the FrancEchino registry.

## Materials and methods

### Patient population

The STCS is a national cohort that has been following all SOT recipients in Switzerland since May 2008 [21]. The structure of the STCS has been described elsewhere [8]. Briefly, lifelong follow-up is mandatory by law for all transplanted patients. Additionally, the majority of patients (>93%) accept to participate in the collection of a full dataset, including clinical data of infectious disease episodes and biological samples [21]. The STCS has been approved by the local Ethics Committee of all participating centers, and all patients gave written informed consent. As of August 2018, 4,869 patients had been included in the STCS.

The FrancEchino registry has been collecting data on AE cases in France since July 1982; it is part of the activities of the French National Reference Center on Echinococcosis, accredited by “Santé Publique France”, the French national agency for disease surveillance. As the declaration of AE cases is not mandatory in France, the registry relies on collaboration with hospital centers, with parasitology laboratories all over the country and on monitoring of pharmacies for the prescription of albendazole to detect incident cases [6]. Until the end of 2018, 803 cases had been included in the registry, of which 369 during the study period. The FrancEchino registry has been approved by the Region ethics committee (CPP) and the National committee on data privacy (CNIL), and all patients gave their informed consent.

### Data collection

We searched for *de novo *cases of AE in SOT recipients in the STCS database and the FrancEchino registry. We included all cases of AE diagnosed in the May 2008 to August 2018 period. We also searched for potentially missing cases in the National Transplantation database of the French Agency of Biomedicine, which had registered 57,905 cases of solid organ transplantations during the study period (2008–2018). Only *de novo* AE in SOT recipients were included. We excluded patients who received a liver transplant to treat an already diagnosed AE before transplantation. Of note, this article follows the international recommendations on echinococcoses terminology [29].

Additional data that were not included in the STCS database or the FrancEchino registry were collected using a case-report form. We collected data on the clinical presentation of AE (including time of onset after transplantation, clinical signs and symptoms), immunosuppressive regimens used at the time of diagnosis, and diagnostic procedures, including imaging and serology results. We also collected data on the treatment of AE, including surgical procedure(s) (complete *vs* incomplete liver resection) and the use of anti-parasitic drugs. Clinical outcomes after the initiation of therapy were assessed by documenting reports of magnetic resonance imaging (MRI), positron emission tomography-computed tomography (PET-CT), and serology titers observed during follow-up. Of note, serologic assays may vary from center to center, depending on their availability at diagnosis and follow-up. The definition and diagnostic performance of the different serologic assays used in this study are summarized in Supplementary Table 1 [30, 31]. Results above the positive threshold for at least one test were used to define positive serology in a given patient.

The PNM classification (P = parasitic mass in the liver, N = involvement of neighbouring organs, and M = metastasis) was used for the initial staging of the disease. This classification is a staging system based on the location and extent of the primary lesion in the liver, the involvement of neighboring organs, and the presence of distant metastasis [17]. During the follow-up, the evolution was classified as “progression” if the imaging showed an increase in size of the lesions, “stabilization” if the size of the lesion did not vary, or as “regression” if the size of the lesion diminished.

### Statistical analysis

We used descriptive statistics to characterize the study population. Categorical variables were described as frequency rates and percentages. Continuous variables were described using mean, median and the range between the minimum and maximum values.

## Results

### Patient population

We identified eight cases of *de novo* AE in SOT recipients in both cohorts, two in Switzerland and six in France during the 10-year study period, which represents 0.01% of the total number of patients with a solid organ transplantation both in France and in Switzerland. It also represents 1.6% of the total number of French patients with AE collected in the FrancEchino database during the same period. The mean age at diagnosis was 54 years and the median time from transplantation to diagnosis of AE was 66 months (range 12–240). Most patients were kidney transplant recipients (*n* = 5); no patients were liver transplant recipients. The majority of patients received a combination of calcineurin inhibitor and mycophenolate mofetil (MMF). The baseline characteristics of the included patients are summarized in Table 1. Two cases have been described in separate case reports [10, 12].

**Table 1:** Baseline characteristics, clinical manifestations and outcomes of the eight SOT recipients with AE.

ID	Sex; F: female; M: male	Age at diagnosis	Transplanted organ	Time from transplant to diagnosis (in months)^a^	Immunosuppressive regimen	Clinical presentation	Extension	PNM stage	Diagnostic delay^b^ (in months)	Comments
1	F	57	Lung	43	Prednisone, MMF, Tacrolimus	Asymptomatic	–	P1N0M0 (I)	1	
2	F	53	Kidney (2×)	12^a^	Prednisone, Tacrolimus	Fever and pain in left hypochondrium	Stomach and esophagus	P1N1M0 (IIIb)	–	
3	M	55	Kidney	91	MMF, Everolimus	Pain in right hypochondrium	–	P2N0M0 (I)	11	Radiological lesions not immediately investigated
4	F	62	Kidney (2×)	240^a^	Prednisone, cyclosporine, azathioprine (switched to MMF)	Asymptomatic	–	P1N0M0 (I)	30	Radiological lesions not immediately investigated
5	F	66	Heart	117	Prednisone, MMF, cyclosporine	Epigastric pain	Head of pancreas	P4N1M0 (IV)	–	
6	F	59	Kidney	57	MMF, Tacrolimus	Asymptomatic	–	P0N0M1 (IV)	12	Initial misdiagnosis of tuberculosis
7	M	36	Kidney	75	Prednisone, MMF, Cyclosporine	Asymptomatic	–	P1N0M0 (I)	3	Initial misdiagnosis of metastasis
8	F	43	Lung	12	Prednisone, MMF, Tacrolimus	Pain in right hypochondrium	–	P1N1M0 (IIIb)	–	

a Time from the first transplantation to diagnosis.

b Time from first evidence of a radiological lesion to final diagnosis. MMF: MycoPhenolate Mofetil.

### Clinical presentation

Out of the eight cases of AE, the primary lesion was located in the liver for seven cases and in the lung in one case (Table 1). In the latter case, the patient also had autosomal dominant polycystic kidney disease (ADPKD), which impaired the radiological identification of additional liver lesions. AE presented with abdominal pain in four patients and was an incidental radiological finding in asymptomatic patients in the other four cases.

AE extended beyond the liver in two patients (pancreas and stomach/esophagus, respectively), but no patients in this case series presented with metastases. Most symptomatic patients presented with high PNM stages [17]: one patient with stage I, two patients with stage IIIb and one patient with stage IV, while most asymptomatic patients presented with low PNM stages (3 patients with stage I and one patient with stage IV).

### Diagnosis

Diagnosis of AE was achieved through a combination of imaging and serology in all cases. A summary of the results of serology and MRI is shown in Table 2. The sensitivity of the screening serology varied: 60% (3/5 cases) for Em2+, 0% (0/1 case) for Em2, 100% (6/6 cases) for EgHF and 75% (3/4 cases) for IHA. Western blot was always positive and allowed for identification at the species level in four cases. In order to identify the onset of infection, retrospective AE serology was performed on stored sera for patient #1 and patient #8: they were negative 43 (Em2+) and 12 months (Em2+ and Eg IHA) prior to the diagnosis of AE, respectively. However, Western blot performed on the same stored sera of patient #8 showed a weak 7 kDa band, compatible with an abortive lesion [10].

**Table 2:** Diagnosis of alveolar echinococcosis (AE) in patients with solid organ transplantation.

Serology (ND: not done)					
ID	Em2+	Em2	EgHF	IHA (titers)	Western blot^a^	MRI^c^
1	Negative	Negative	Positive	ND	Positive for AE^b^	Several nodular lesions with T2 hyper signal. Imaging not typical for AE. Serologies were performed due to a high suspicion.
2	ND	ND	ND	ND	Positive for AE^b^	Multiple confluent cystic lesions. Imaging not typical for AE. Abscess-like appearance.
3	Positive	ND	Positive	ND	Positive bands 7 + 26 − 28	Centimetric hepatic lesion in T2 hypersignal, with several microcysts.
4	Positive	ND	Positive	Positive (1:640)	Positive bands 7 + 26 − 28	Lesions atypical for AE. Tumor-like appearance
5	Negative	ND	Positive	ND	Positive bands 16 + 26 − 28	MRCP: 2.6 cm lesion of the head of the pancreas in T2 hypersignal. Suspicion of pancreatic cancer
						Liver MRI: three centimetric lesions with peripheral microcysts
6	ND	ND	Positive	Positive (1:320)	Positive bands 7 + 26 − 28	Liver MRI only showed cysts (patient with ADPKD)
7	ND	ND	ND	Positive (1:1280)	Positive bands 7 + 26 − 28	Centimetric T2 lesion with a metastatic appearance
8	Positive	ND	Positive	Negative (<1:40)	Positive bands 7, 16, 18, 26 − 28	Voluminous 8.5 cm lesion, with several infra-centimetric peripheral lesions. Tumoral appearance.

a Bands at 7 and 26–28 kDa are specific for *Echinococcus*, bands at 16 and 18 kDa are specific for *Echinococcus multilocularis *[23].

b The molecular weights of the positive bands were not reported.

c Descriptions and conclusion from the radiology reports, when available. MRI: Magnetic Resonance Imaging; MRCP: Magnetic Resonance Chlolangio-Pancreatography. ADPKD: Autosomal Dominant Poly- cystic Kidney Disease.

All patients had a liver MRI performed. Despite AE having an atypical radiological (US and/or CT-scan) presentation in five cases (a tumor-like image in most cases), the correct diagnosis was suspected on MRI images in seven out of the eight cases. The patients had lesions with a typical AE appearance: microcystic with hypersignal on T2-weighted MR images [24]. For patient #6, the interpretation of the liver MRI was complicated by the ADPKD. The correct diagnosis was suspected based on histopathology of a lung biopsy and confirmed by *Echinococcus* spp. Western blot serology.

**Figure 1:**
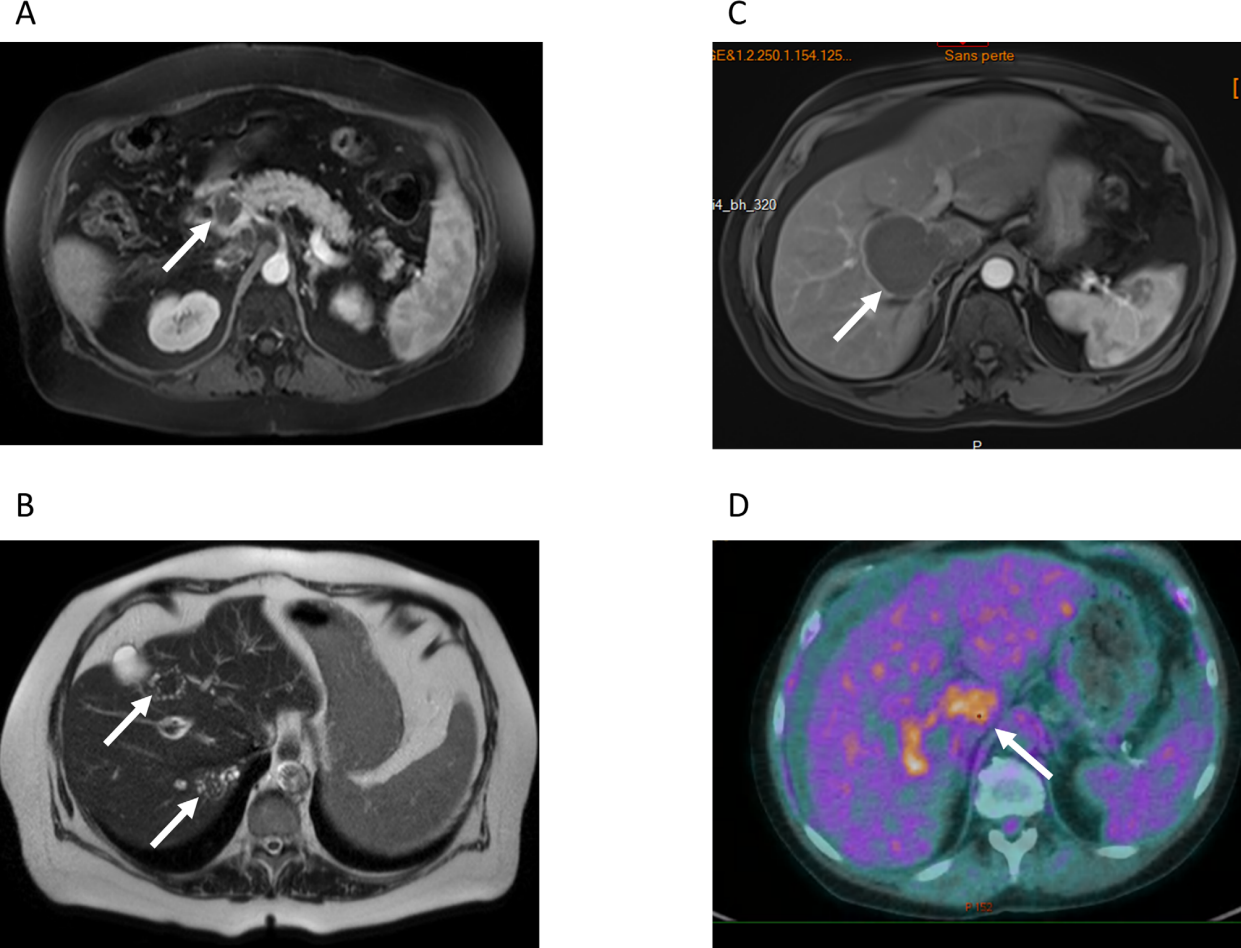
*Radiological findings. *A) and B) MRI of patient #5 A) T1-weighted MRI of patient #5. White arrow: lesion in the head of the pancreas. This lesion was mistakenly interpreted as pancreatic cancer. B) T2-weighted, the white arrows point at the typical microcysts in T2-hypersignal. C) T1-weighted MRI of patient #4, showing a large well-delimited lesion tumor-like lesion, with invasion of the right portal branch D) PET-CT of patient #4, showing intense metabolic activity at the periphery of the lesion.

Diagnosis of AE in asymptomatic patients was often delayed, with a mean delay of 11.5 months between the finding of the radiological lesion and the final diagnosis, as compared to 2.75 months in symptomatic patients. For two asymptomatic patients, the diagnostic delay was due to a misdiagnosis of tuberculosis and metastatic cancer, respectively (Table 1 and Fig. 1). Of interest, two patients had imaging without any lesion suggestive of AE 36 month (Patient #1) and 26 months (Patient #2) prior to the diagnosis of AE. For patients #3 and #4, the AE lesion was already present on previous imaging dated 11 and 40 months prior, respectively, before the diagnosis (but after transplantation), and had not been investigated.

### Treatment and outcomes

The median follow-up duration after diagnosis of AE was 36.5 months (range 0–49). Therapy and outcome are summarized in Table 3. All but patient #1 (who died of peri-operative complications and was not treated before surgery) received albendazole at an initial dose of 400 mg twice daily. No patients reported adverse events that led to discontinuation of the drug. Of note, drug-drug interactions with calcineurin inhibitors or mTOR inhibitors were not reported.

**Table 3:** Treatment, follow-up and outcome of AE in patients with solid organ transplantation.

	Treatment and outcome			Follow-up		
ID	Surgical intervention (months from diagnosis)	Albendazole	Outcome	Follow-up (months)	Follow-up serology	Follow-up imaging
1	Incomplete resection (0 m)	No	Deceased^a^	0	–	–
2	Incomplete resection (0 m)	400 mg bid	Stabilization	43	Negative at 19 and 42 months b	MRI at 32 and 43 months: stabilization
3	No	400 mg bid, increased to 400 mg tid (due to the progression)	Progression	49	EgHF ELISA (at 17 and 30 m): positive, titers decreasing	PET-CT (at 32 and 49 m): persistence of metabolic activity; progression
					AE serology b (at 17 and 30 m): positive, titers increasing	MRI at 44 months: progression
					Western blot (at 17 and 30 m): positive	
4	Incomplete resection (31 m)	400 mg bid, increased to 400/600 mg	Deceased^a^	31	–	–
5	No	400 mg bid	Deceased	5	–	–
6	Incomplete resection^c^ (2 months prior diagnosis)	400 mg bid	Stabilization	20	Serology^b^ titers decreasing at 18 and 20 months	Chest CT-scan at 6 and 12 months: no new lesion
7	No	400 mg bid	Stabilization	48	Eg IHA and ELISA^b^ at 3, 6, 18, 24, 30, 36 and 48 months: decreasing titers (rebound of IHA at 30 m, then decrease)	MRI at 18 and 48 months: stabilization
8	Complete resection (14 m)	400 mg bid, decreased to 200 mg bid (supra-therapeutic concentration)	Regression	42	EgHF ELISA (0 and 4 m): decreasing titers	MRI (9 m): no lesion
					Em2+ ELISA (0, 4, 6 m): decreasing titers; negative at 42 m	PET-CT : 1 month: residual metabolic activity; 13 and 42 m : no metabolic activity
					Eg IHA (0, 4 and 6 m): neg, neg, pos	
					Western blot (0, 4 and 6): pos, neg, pos	

a Died of peri-operative complications;

b The type of assay or the antigen is unknown. AE: Alveolar Echinococcosis; IHA: Indirect HemAgglutination.

c The resection was performed for diagnostic purpose. MRI: Magnetic Resonance Imaging; CT: Computed Tomography; PET-CT: Positron Emission Tomography associated with Computed Tomography.

Five out of eight patients underwent liver surgery. Complete resection was achieved in only one case (patient #8). Only partial resection could be achieved for the other four patients. Two out of the five patients who underwent surgery died of peri-operative complications (patient #1 and patient #4). Patient #1 died after acute liver failure and disseminated intravascular coagulation of unknown origin, leading to widespread bowel ischemia. Patient #4 was initially treated with albendazole alone for 31 months: surgical resection was attempted to avoid the progression of AE lesions in prevision of a scheduled increase in the immunosuppressive treatment for a third kidney transplantation. The exact cause of death is unknown. One additional patient died five months after diagnosis, while in rehabilitation after a stay in an intensive care unit due to septic shock in the context of cholangitis (patient #5). Cholangitis was likely a complication of the parasitic mass in the head of the pancreas (Fig. 1). None of the three patients receiving anti-parasitic therapy alone died during follow-up. AE was considered cured in the case where complete resection could be achieved (patient #8). AE was considered to be stable in four other cases and progressed in one.

**Figure 2:**
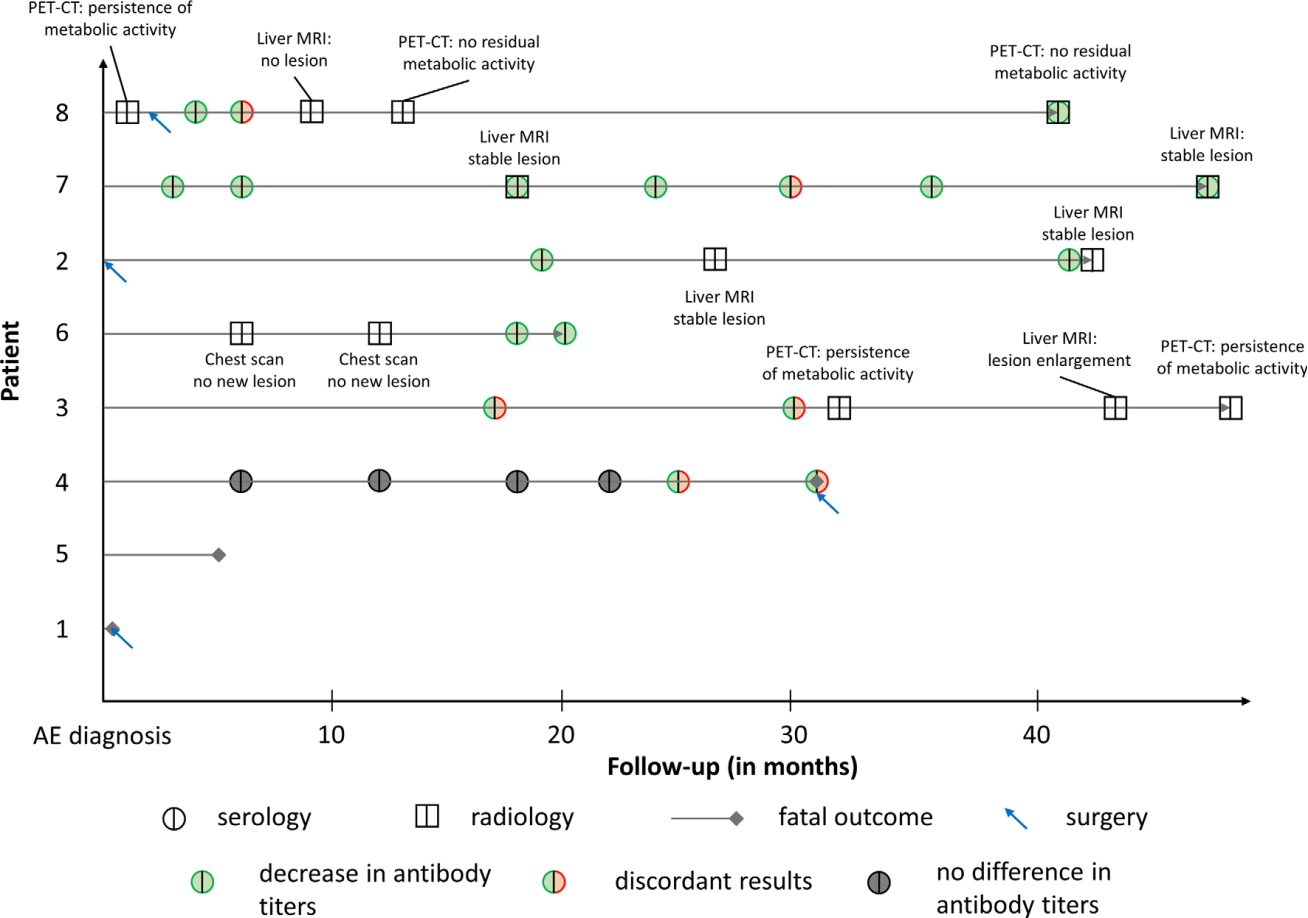
*Summary of the patients’ follow-up after AE diagnosis in solid organ transplant recipients*. “Discordant results” indicates discordant results in serological assays performed at the same time.

The detailed follow-up of patients is shown in Table 3 and Figure 2. All surviving patients had both serological and radiological follow-ups. Follow-up using liver MRIs showed stabilization in two cases, regression in one and progression in one. Follow-up using PET-CT showed absence of metabolic activity in one case and increase in the others. Patient #6 had two follow-up chest scans that did not show any recurrence.

## Discussion

This study highlights the difficulty to diagnose and treat AE in SOT recipients. The interpretation of radiological images is complicated by atypical presentations and the usual serology tests are not as sensitive as in immunocompetent patients. However, *Echinococcus spp. *Western blot serology was shown to be reliable and should be the preferred alternative to confirm AE in SOT recipients. In addition, high mortality associated with surgery, failure to achieve complete resection in all but one case, and good tolerance of albendazole, all speak in favor of a conservative approach to treat these patients.

In this series, the incidence of AE in SOT recipients with chronic immunosuppression was estimated to be 0.01% of the total number of patients with SOT in France and Switzerland. Data from the FrancEchino cohort suggest that this incidence is approximately 20-fold higher than in subjects without SOT. However, given the low number of patients included and because a potential bias towards earlier diagnosis due to a higher number of radiological examinations performed in our cohort, we cannot robustly confirm the findings of previous studies highlighting a faster course of AE in immunocompromised patients [7, 14]. Interestingly, AE was an incidental diagnosis in 50% (4/8) of patients of this case series. While older studies reported a higher percentage of symptomatic patients (70%) in the general population [18, 25], the clinical presentation of more recent cases of AE was closer to that observed in this case series, with only 41% of patients being symptomatic at diagnosis (data from the FrancEchino registry in the same period). The asymptomatic patients of this case series often had considerable diagnostic delay. In two cases (patients #3 and #4), this delay was due to the lack of initial investigations despite hepatic lesions being reported in the radiologist reports. As the radiological examinations were performed for suspicion of renal artery stenosis (in patient #3) and in a pre-transplant evaluation before a 2nd renal transplant (in patient #4), the abnormal images were likely neglected as this was not the main concern. Of note, these two patients also had a more severe outcome. Half of the patients (50%) had localized stages (stage I–II) at the time of diagnosis, unlike what is reported for immunocompetent patients, but common in immunocompromised patients [7, 17]. Interestingly, no patients presented with distant foci of infection. This is likely a consequence of a closer follow-up of SOT recipients, as suggested by the shorter diagnostic delay in those patients than in non-immunocompromised patients presenting with atypical symptoms [25]. It could also be explained by patients becoming symptomatic faster due to more rapid growth of the lesions. The same reasons likely explain the biphasic distribution of stages, with the disease diagnosed at either low or high stages. The short median interval between the diagnosis and the radiological proof of absence of AE 36 and 26 months (patient #1 and #2, respectively) prior to diagnosis bring indirect evidence of faster development in immunocompromised patients, although these data need to be taken with caution given the low number of patients included. As was observed in other patients with therapeutic immune suppression including after liver transplantation for AE [2, 7], long-term prednisone, present in 6/8 patients could be a risk factor for the development of AE lesions in SOT patients. However, the small sample size in this study precludes definitive conclusions on the risk of developing AE associated with specific immunosuppressive drugs. Interestingly, no AE cases were found in liver transplant recipients, despite the liver being an organ frequently transplanted. This may be due to lower immunosuppression in these patients [2]. Alternatively, it could be due to the incidental cure of latent AE lesions by liver transplantation. Such lesions might be reactivated by the immunosuppression when another organ is transplanted, as may have been the case for patient #8 whose pre-transplant serum exhibited a 7 kDa band at Western Blot, suggesting pre-transplantation exposure to *E. multilocularis *without apparent lesions. Altogether, this case series shows that a high level of suspicion is warranted in case of unexplained hepatic lesions in SOT recipients, particularly when they live or have lived in an endemic region. In these cases, a hepatic MRI and an *Echinococcus *spp. Western blot serology should be obtained as the combination of both was shown to have good diagnostic performance.

Our case series also confirms the lower diagnostic performance of the AE screening serology reported in immunocompromised patients in previous studies [7]. Em2+, usually reliable with a reported sensitivity of 97% [15, 19] performed poorly and should be interpreted with caution in this setting. However, the serology based on less specific antigens performed better, in line with previous studies [3, 22]. Indeed, in patients who underwent liver transplantation in the context of AE, an ELISA using *E. granulosus* antigens was better at predicting recurrence than an Em2 ELISA [3]. This may be due to higher immunogenicity of the *Echinococcus* genus-related antigens that may elicit a humoral immune response despite the immunosuppression, when the more specific but less immunogenic antigens fail to do so. Since the standard serology tests were often negative at diagnosis, the choice of serological assays was often limited for the follow-up, and the serology results were difficult to interpret. The recommended follow-up serology using Em18 [16, 27] was not used for any patient in this case series: it could be interesting to assess its performance in immunocompromised patients in a future study. However, its high specificity for *E. multilocularis* may raise doubts about its usefulness in this situation which could conversely be an appropriate indication for the use of the more sensitive and qualitative Western Blot.

In this case series, surgical interventions had a surprisingly high mortality (2/5 patients) and a low rate of success: only one complete resection could be achieved (patient #8). This high mortality is probably due to these patients being more fragile than those usually treated for AE. Preoperative assessment should accordingly be thorough in these patients: according to the international recommendations, surgery should only be attempted if complete resection is achievable, and a multidisciplinary discussion of each case before surgery should be the rule. In addition, preoperative treatment with albendazole is highly recommended [4, 28, 30], especially since its efficacy in immunosuppressed patients is well documented [2]. In this series of SOT recipients, albendazole was as effective as in other studies in immunosuppressed patients [2, 3, 7, 20, 28], allowing regression or stabilization in all but one case. In that case, AE progressed despite blood concentrations of albendazole in the therapeutic range, prompting an increase in dosage from 400 mg bid to 400 mg tid. The treatment was well tolerated, which contrasts with the previous report on AE in immunocompromised patients [7] where side effects seemed to be more frequent. This difference may be due to the small population of patients of this study, but also to marked differences in associated drugs between patients with SOT and patients with malignant tumors or chronic inflammatory diseases. The inability to obtain a complete resection, the high mortality associated with surgery and the efficacy of albendazole speak in favor of a conservative approach to treat AE in SOT recipients. Albendazole therapy should be systematically initiated and surgery should be attempted only if complete resection can be attained with a safe procedure.

In conclusion, despite limitations due to the small sample size, this case series suggests that the incidence of AE is higher in SOT than in the general population of endemic regions; it suggests that the parasitic disease might be due to the reactivation of latent microscopic lesions through immunosuppression; it also stresses the diagnostic efficacy of MRI and Western blot for the diagnosis of AE in SOT patients. Diagnostic delays should be avoided by keeping a high index of suspicion in case of a hepatic lesion, particularly in a setting of immunosuppression that favors faster growth of AE lesions. Given the efficacy and good tolerance of albendazole and the high mortality of surgery in this series, any decision regarding interventional procedures in SOT recipients should be discussed by a multidisciplinary team.

## Supplementary material

The supplementary material of this article is available at https://www.parasite-journal.org/10.1051/parasite/2023008/olm. *Supplementary Table 1*: Summary of the published performance of the different serologic assays used in this study.

## Appendix

### List of active members of the Swiss Transplant Cohort Study (STCS):

Patrizia Amico, Andres Axel, John-David Aubert, Vanessa Banz, Beckmann Sonja, Guido Beldi, Christoph Berger, Isabelle Binet, Pierre-Yves Bochud, Sanda Branca, Heiner Bucher, Thierry Carrel, Emmanuelle Catana, Yves Chalandon, Sabina de Geest, Olivier de Rougemont, Michael Dickenmann, Joëlle Lynn Dreifuss, Michel Duchosal, Thomas Fehr, Sylvie Ferrari-Lacraz, Christian Garzoni, Paola Gasche Soccal, Christophe Gaudet, Déla Golshayan, Karine Hadaya, Jörg Halter, Dimitri Hauri, Dominik Heim, Christoph Hess, Sven Hillinger, Hans Hirsch, Patricia Hirt, Günther Hofbauer, Uyen Huynh-Do, Franz Immer, Michael Koller (Head of the data center), Bettina Laesser, Brian Lang, Roger Lehmann, Alexander Leichtle, Christian Lovis, Oriol Manuel, Hans-Peter Marti, Pierre Yves Martin, Michele Martinelli, Katell Mellac, Aurélia Merçay, Karin Mettler, Nicolas Mueller (Chairman Scientific Committee), Antonia Müller, Thomas Müller, Ulrike Müller-Arndt, Beat Müllhaupt, Mirjam Nägeli, Manuel Pascual (Executive office), Klara Posfay-Barbe, Juliane Rick, Anne Rosselet, Simona Rossi, Silvia Rothlin, Frank Ruschitzka, Urs Schanz, Stefan Schaub, Aurelia Schnyder, Macé Schuurmans, Federico Simonetta, Katharina Staufer, Susanne Stampf, Jürg Steiger (Head, Excecutive office), Guido Stirniman, Ueli Stürzinger, Christian Toso, Christian Van Delden (Executive office), Jean-Pierre Venetz, Jean Villard, Julien Vionnet, Madeleine Wick (STCS coordinator), Markus Wilhlem, Patrick Yerly.

### List of contributors to the FrancEchino network:

Amiens: Nguyen-Khac Eric, Rodriguez Marc Antoine, Sarba Ruxandra, Tilmant Marion; Angers: De Gentile Ludovic; Annecy: Janssen Cécile, Juget-Pietu Florence, Maillet Mylène, Piet Emilie, Philippe Laure, Tolsma Violaine, Vitrat Virginie; Besançon: Alby-Lepresle Blandine, Anxionnat Raphaël Bartholomot Brigitte, Beurton-Chataigner Isabelle, Blagosklonov Oleg, Bresson-Hadni Solange, Brumpt Eléonore, Chirouze Catherine, Delabrousse Eric, Di Martino Vincent, Felix Sophie, Giraudoux Patrick, Grenouillet Frédéric, Heyd Bruno, Knapp Jenny, Koch Stéphane, Millon Laurence, Montange Damien, Raoul Francis, Richou Carine, Thevenot Thierry, Turco Célia, Vanlemmens Claire, Verhoeven Weil Delphine , Vuitton Dominique; Béthune: Nguyen Sophie; Bordeaux: Bernard Pierre-Henri, Desclaux Arnaud, Duvignaud Alexandre; Bourges: Guimard Yves; Brest: Grall-Jezequel Anne, Rogeaux Olivier; Caen: Altieri Mario, Baldolli Aurélie; Chalon Sur Saône: Fillion Aurélie; Chambéry: Al Nassan Irchid, Rogeaux Olivier; Clamart: Popa Ingrid; Clermont-Ferrand: Abergel Armand, Beytout Jean, Nourrisson Céline, Poirier Philippe, Reymond Maud; Colmar: Denis Bernard, Topolscki Amalia; Contamine-Sur-Arve: Challan Belval Thibaut, Huet-Penz Floriane, Huguet Dominique, Lacoste Marie; Créteil: Leroy Vincent; Dijon: Besancenot Jean-François, Bruel Bruno, Dalle François, Gonnot Maxime, Hillon Patrick, Latournerie Marianne, Minello Anne, Valot Stéphane; Epinal: Auburtin Marc, Camara Mory, Schuhmacher Marie-Hélène; Grenoble: Chirica Mircea, Costentin Charlotte, Faure-Cognet Odile, Goutier Sandrine, Letoublon Christian, Pavese Patricia Wintenberger Claire; La Rochelle: Pouget-Abadie Xavier; Lille: Faure Karine, Louvet Alexandre; Lyon: Dumortier Jérôme, Guillaud Olivier, Guillet Marielle, Partensky Christian, Rabodonirina Meja, Wallon Martine; Marseille: Ratone Jean-Philippe, Rebaudet Stanislas; Metz: Chatelain Eric, Johann Marc; Mulhouse: Claude Pierre, Sondag Daniel; Nancy: Bensenane Oussalah Mouni, Bronowicki Jean-Pierre, Frentiu Emilia, Lefeve Benjamin, Machouart Marie; Nantes: Archambeaud Isabelle; Orléans: Thebault Baudoin; Paris: Belan Martin, Borghi Scoazec Giovanna, Bouree Patrice, Chopinet Sophie, Farges Olivier, Gontier Hugues, Jaureguiberry Stéphane, Kerneis Solen, Laffont Emmanuel, Parlati Lucia, Piarroux Renaud, Plessier Aurélie, Samuel Didier, Sogni Philippe, Sommacale Danièle; Reims: Chemla Cathy, Rhaiem Rami, Thiefin Gérard, Villena Isabelle; Rennes: Autier Brice, Beldeyrou Marion, Boudjema Karim; Roanne: Murat Jean-Benjamin; Rouen: Gargala Gilles; Saint-Dizier: Delanoe Christophe; Saint-Flour: Kallita Mohammed; Saint Ouen L’aumône: Debruyne Monique; Strasbourg: Abou-Bacar Ahmed, Goichot Bernard, Hansmann Yves, Lefebvre Nicolas; Thonon Les Bains: Mourey-Epron Catherine; Toul: Watelet Jérôme; Toulouse: Alric Laurent, Lelievre Lucie; Trévenans: Winkfield Betsy; Valenciennes: Quinton Jean-François; Veoul: Monet Matthieu; Villejuif: Sa Cunha Antonio.
