# Supplementary material for: Alveolar echinococcosis in solid organ transplant recipients: a case series from two national cohorts
Source: Parasite. 2023 Mar 31;30:9. doi: 10.1051/parasite/2023008 (PMC10069401; doi:10.1051/parasite/2023008)
Supplement: Supplementary Table 1: — Summary of the published performance of the different serologic assays used in this study. [file parasite-30-9-s1.pdf]

Supplementary Table 1: Summary of the published performance of the different serologic assays used in this study

Serology	Sensitivity	Specificity	Commentary	Reference
Em2 ELISA	89%	98%	Uses Em2 antigen (from *E. multilocularis* laminated layer), an antigen which may persist in non-viable AE lesions	[15]
Em2+ ELISA	97%	90%	Uses a combination of the Em2 antigen and a recombinant II/3-10 protein (antigen considered to be a marker of metacestode viability)	[15]
EgHF	97%	61%	Uses heterologous antigen (*E. granulosus s.l.* fluid).	[15]
Indirect Hemagglutination Assay (IHA)	74% (1:320)<br>94% (1:80)	ND	Uses heterologous antigen (*E. granulosus s.l.* fluid). Sensitivity is highly improved by using a 1/80 dilution threshold [1].	[1,23]
Western blot	97%	Varies between 88 – 100%.<br>Depends on the MW of the observed bands [a].	Uses whole *E. multilocularis* metacestode antigenic extract. Depending on the detected bands, allows the differentiation at the species level.	[23]

[a] MW: molecular weight; bands at 7 and 26-28 kDa are specific for *Echinococcus*, bands at 16 and 18 kDa are specific for *Echinococcus multilocularis*
